# Impact of Bedside Handover on Patient Perceptions and Hospital Organizational Outcomes: A Systematic Review

**DOI:** 10.1155/jonm/3803491

**Published:** 2025-07-21

**Authors:** Chiara Daicampi, Mayra Veronese, Elisabetta Cesaro

**Affiliations:** ^1^Department of Medicine, University of Padua, Padua, Italy; ^2^Department of Health Professions, University Hospital of Padua, Padua, Italy; ^3^Department of Cardiac, Thoracic, Vascular Sciences and Public Health, University of Padua, Padua, Italy

## Abstract

**Background:** Nursing handover is a critical component of healthcare, ensuring continuity of care and patient safety. Bedside handover, conducted at the patient's bedside during shift changes, has been proposed as a strategy to enhance communication among healthcare professionals, increase patient involvement, and optimize hospital efficiency. However, concerns persist regarding privacy, time constraints, and the quality of information exchanged.

**Aim:** This systematic review aims to assess the impact of bedside handover on patient outcomes and hospital organizational performance, evaluating both its benefits and challenges.

**Methods:** A systematic review was conducted following PRISMA guidelines. Quantitative and qualitative studies published between 2004 and 2024 were retrieved from PubMed, Scopus, and CINAHL. The methodological quality of the selected studies was assessed using the Joanna Briggs Institute (JBI) appraisal tools. Data were synthesized narratively.

**Results:** Out of 6396 initially identified records, 14 studies met the inclusion criteria, but one was excluded following quality appraisal due to low methodological quality. Therefore, 13 studies were included in the final review. Bedside handover was associated with significant benefits for patients, including improved perceived safety, satisfaction, reduced anxiety, and active involvement in their care. From an organizational perspective, bedside handover led to reduced handover duration, decreased shift overtime, and fewer nurse call requests, contributing to cost savings. However, concerns about patient privacy and variability in implementation practices were identified as potential barriers.

**Conclusion:** Bedside handover represents a valuable opportunity to improve patient-centered care and hospital efficiency. Despite certain challenges, its advantages outweigh its drawbacks. Effective implementation requires addressing privacy concerns and providing adequate training for healthcare professionals to ensure consistency and adherence to best practices.

## 1. Introduction

Nursing handover is a critical component of healthcare, involving the transfer of patient information and responsibilities between nurses during shift changes [1, 2]. This process is essential for maintaining continuity and ensuring the quality of patient care by providing incoming nurses with updated information on patients' conditions, treatment plans, and significant developments from the previous shift [3, 4]. When performed effectively, nursing handovers enhance patient safety, support the delivery of high-quality care, and improve overall healthcare efficiency [5, 6]. Additionally, handovers facilitate clear communication among healthcare providers and encourage patient involvement in their care, which can help prevent errors such as miscommunication or medication-related mistakes [4, 7–9]. Conversely, poor handover practices have been associated with adverse outcomes, including higher rates of falls and pressure injuries [8].

An integrative review by Bakon et al. [10] identifies four primary styles of nursing handover: verbal bedside handover, tape-recorded handover, verbal handover, and written handover. This highlights the evolution of handover practices in response to patient safety concerns and the need for effective communication [10]. Among these, bedside handovers have demonstrated value in enhancing patient involvement and safety by allowing patients to actively participate in the process [11, 12]. This approach enables patients to clarify inaccuracies in their care, improve their understanding and satisfaction, and foster a collaborative environment that strengthens the overall effectiveness of handovers [12, 13]. Furthermore, Forde et al. [14] underscore the importance of evaluating bedside handover practices, noting that factors such as nurse competence and confidence significantly influence the success of these processes. Studies also suggest that nurses' perceptions of handover practices are crucial; nurses who feel supported and adequately trained are more likely to perform effective handovers [6, 15].

Despite its advantages, bedside handover presents challenges. Some nurses report discomfort with sharing sensitive information in the presence of patients and family members, as highlighted by Tobiano et al. [16]. This concern is echoed by Bukoh and Siah [17], who emphasize that confidentiality issues can deter full adoption of bedside handover practices [17]. Additionally, variability in handover practices across different healthcare settings can lead to inconsistencies in patient care, as observed by Chaboyer et al. [18] in their case study on bedside nursing handover.

Numerous reviews have examined nursing handover practices, including bedside handovers. These reviews have primarily focused on topics such as the outcomes of handover interventions [8], the variable duration of bedside handovers per patient (ranging from 83 to 204 s) [19], patients' perceptions [20], and the meaning and challenges of bedside handovers [21]. Although significant research has been conducted on these topics [8, 19–21], a comprehensive understanding of the impact of bedside handovers on both patient and organizational outcomes remains lacking.

This systematic review aims to address this gap by examining the effects of bedside handovers in hospital settings. Conducted at the patient's bedside during shift changes, these handovers are proposed to enhance communication, improve patient safety, and increase healthcare efficiency. By synthesizing evidence, this review evaluates the extent to which bedside handover practices contribute to improved patient care, staff efficiency, and operational performance in hospital environments. The guiding research question for this review is: What are the effects of bedside handover on patient outcomes and hospital organizational performance?

## 2. Methods

### 2.1. Study Design

This systematic review was guided by the Preferred Reporting Items for Systematic Reviews and Meta-Analyses (PRISMA) statement [22]. A predefined protocol was designed and registered in the International Prospective Register of Systematic Reviews (PROSPERO) database. The registration number was CRD42024616225.

### 2.2. Search Strategy

The search strategy was developed using Medical Subject Headings (MeSH) terms and relevant keywords, based on the PIOs framework. Specifically, the Population (P) included adult hospitalized patients (≥ 18 years) and healthcare personnel; the Intervention (I) considered was bedside handover; the Outcomes (O) focused on clinical results and patient satisfaction, including aspects such as quality of care, safety, error reduction, patient-centered care, the therapeutic relationship, and communication; and finally, the Study types (S) to be selected were defined accordingly (Supporting Table 1).

The databases explored included PubMed, Scopus, and CINAHL, using specific keywords and search terms adapted to each database, combined through the use of Boolean operators (“AND”, “OR”). The complete search strategy is available in Supporting Table 2. The search was carried out in November 2024. Studies were selected based on the following inclusion criteria: (a) primary quantitative and qualitative studies; (b) full text available; (c) published in English; and (d) published between January 2004 and November 2024. The 20-year time frame was chosen to ensure a comprehensive overview of the most relevant and recent developments of bedside handover practice over time. This period captures both the early implementation efforts following the promotion of patient-centered care models and more recent studies reflecting current clinical practice, technologies, and healthcare policies. Studies focused on pediatric setting, published before January 2004, literature reviews, books or reports, research protocol, and gray literature were not considered. All documents were retrieved via the abovementioned strategies and transferred to Rayyan software for screening and extraction.

### 2.3. Quality Appraisal

The Joanna Briggs Institute (JBI) quality appraisal tools for quasi-experimental studies, cross-sectional studies, and qualitative studies were adopted to evaluate the quality of the selected studies and assess the risk of bias (RoB). The methodological assessment tools [23] included eight questions for cross-sectional studies, nine questions for quasi-experimental studies, and ten questions for qualitative research. Each question had four possible responses: YES, NO, UNCLEAR, or NOT APPLICABLE. These tools facilitated the evaluation of the methodological quality of each study and determined the extent to which biases in design, conduct, and analysis were addressed [23].

The methodological quality of all included studies was independently assessed by two reviewers (C.D.; E.C.). Any discrepancies between reviewers were resolved through discussion with a third reviewer (M.V.) until consensus was reached. In instances of doubt or disagreement, consensus was achieved through discussion. The quality assessment was further enhanced by categorizing the methodological quality of studies based on the percentage of criteria satisfied, as suggested by Pieper et al. [24]: studies with ≥ 80% of criteria satisfied were rated as high quality, 50%–79% as moderate quality, and < 50% as low quality. This combined approach enabled a more in-depth analysis, interpretation, and evaluation of the findings of the selected studies.

One study was excluded after the quality appraisal phase, as it met less than 50% of the assessment criteria and was therefore rated as low quality. The decision to exclude this study was made to ensure the methodological rigor and reliability of the findings presented in the synthesis. The final number of studies included in the review was thirteen.

### 2.4. Data Extraction and Synthesis

Data extraction was independently carried out by two reviewers (C.D.; E.C.) using the Rayyan platform, adopting the JBI data extraction tool for systematic review [25].

The authors summarized each selected study by describing its authors, year, study design, country, setting, population, aim, intervention or measurement, main findings, and quality of study using a descriptive approach. All extracted data were checked for accuracy by a third reviewer (M.V.). Discrepancies in the extracted data were discussed between the two reviewers or adjudicated by a third reviewer if necessary. A narrative synthesis of the findings from included studies was presented. A narrative synthesis was conducted following the guidance developed by Popay et al. [26] for narrative synthesis in systematic reviews. The process involved organizing the findings thematically, exploring relationships within and between studies, and developing an overall narrative to summarize and interpret the patterns emerging from the data. Key characteristics and findings of included studies were compared and contrasted to identify similarities, differences, and trends across the evidence base.

### 2.5. Ethical Considerations

For this systematic review, obtaining an opinion from the Ethics Committee was not required since its nature was considered secondary. The problem formulation process was conducted with meticulous adherence to the principles of clarity, objectivity, and precision, aiming to achieve substantial results related to interventions and care within the scope of practice.

## 3. Results

### 3.1. Search Results

**Figure 1:**
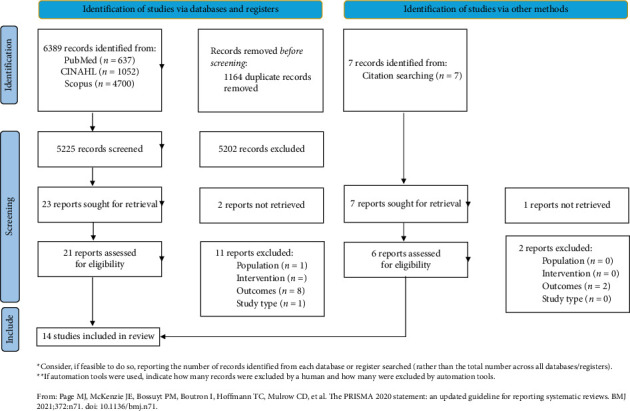
PRISMA 2020 flow diagram for new systematic review which included searches of database, registers, and other sources.

The initial search identified 6389 records. After removing 1164 duplicates, 5225 unique records remained and were independently and blindly screened by two researchers (C.D.; E.C.) during the initial review of titles and abstracts. Following this step, the full texts of 21 studies were assessed by the same reviewers, resulting in the selection of 10 studies. Additionally, 7 records were manually identified from the reference lists of the assessed articles, and after full-text screening, 4 of these were included. In total, 14 studies were included in the review before the quality evaluations [15, 27–39]. Any ambiguities or disagreements regarding the relevance of a study were resolved through consultation with a third researcher (M.V.) [40]. The study selection process is summarized in Figure 1.

### 3.2. Quality Appraisal and RoB of the Included Studies

As illustrated in Supporting Tables 3, 4 and 5, overall, the quality of the studies was found to be low to high. Two mixed-method studies [27, 28] were evaluated separately for their qualitative and quasi-experimental components using two different appraisal tools. Five qualitative studies [28, 29, 33, 36, 39] and two quasi-experimental studies [15, 37] were rated as high quality, representing more than 35% of the total studies. A total of eight studies (57%) were rated as moderate quality: one cross-sectional study [32], one qualitative study [27], and six quasi-experimental studies [27, 28, 30, 34, 35, 38]. Only one qualitative study [31] was rated as low quality and was therefore excluded from the review. Thus, 13 studies remained in the review [15, 27–30, 32–39].

All the included studies demonstrated congruity, meaning there was alignment between the research objectives, the methodological design, data collection techniques, analysis methods, and the interpretation of results. This was assessed using the JBI critical appraisal checklists, which include specific items evaluating consistency between these domains [23].

### 3.3. Study Characteristics

Table 1 summarizes the key characteristics of the included studies. The included studies were published from 2012 to 2019. Regarding the study design, two were mixed-method studies [27, 28], one was cross sectional study [32], five were longitudinal studies [15, 30, 34, 35, 38], one was a one-group pre- and postintervention study [37], and four were qualitative studies [29, 33, 36, 39].

### 3.4. Description of the Population

The studies included are focused exclusively on healthcare workers and adult patients hospitalized in clinical settings. A total of 1662 patients and 824 healthcare workers were analyzed across 13 studies, with sample sizes ranging from 8 to 964 participants per study.

Among the included studies, several were conducted in more than one clinical setting [15, 34, 36, 37]. Specifically, six involved an acute ward [27–29, 33, 36, 37], one focused on oncology patients [32], one a trauma unit [30], one an emergency department [39], one study was conducted in a community hospital [35], and two in a maternity ward [36, 37]. Additionally, two studies addressed medical rehabilitation settings [15, 34], two focused on geriatric settings [15, 34], and five were conducted in surgical wards [15, 34, 36–38] (Table 1).

Geographically, five studies were carried out in Europe [15, 29, 32–34], four studies were conducted in Australia [28, 36, 37, 39], and four were performed in the USA [27, 30, 35, 38] (Table 1).

### 3.5. Organizational Outcome

As summarized in Table 2, bedside handovers are associated with significant organizational benefits, including improvements in staff compliance, reductions in handover duration and overtime hours, cost savings, decreased call light usage and nursing case tasks, and documentation completion.

For instance, Kerr et al. [39] found a nonsignificant decrease in handover duration 12 months after the implementation of bedside handover (from 33.5 min to 30.6 min). Scheidenhelm and Reitz [35] reported that nurse compliance with bedside handovers improved notably after the implementation of targeted training and simulation exercises in obstetric and medical-surgical units. This underscores the importance of preparation and practice in enhancing adherence to handover protocols. Malfait et al. [34] found substantial variability in handover durations before and after the introduction of bedside handovers. Initially, handovers lasted between 6 and 15 min, with individual patient handovers taking anywhere from 75 to 180 s. Following the implementation of decentralized handovers and the ISBARR (Introduction, Situation, Background, Assessment, Recommendation, and Read-back) framework, the duration of handovers per patient ranged from 83 to 204 s, while overall overtime was significantly reduced. Before the intervention, seven out of twelve wards reported frequent overtime due to lengthy handovers. After adopting the new model, most wards successfully eliminated overtime, except for one where challenges in implementing ISBARR led to increased patient handover times despite an overall reduction in handover duration. Similarly, Cairns et al. [30] highlighted the positive impact of bedside shift reporting on overtime reduction. Their findings revealed a decrease of 913 overtime minutes—equivalent to roughly 10 min per day—which translated into annual savings of $95,680 to $143,520. These savings accounted for approximately 23% of the salary budget in the pilot unit, illustrating the economic advantages of bedside shift reporting in curbing overtime-related expenses.

Another notable outcome of bedside handovers was a reduction in call light usage. With nurses more readily accessible to patients during handovers, call light usage decreased by 33% in the morning and 38% in the evening, reflecting an improvement in patient–nurse interaction and responsiveness [30].

Kerr et al. [39] also found significant improvements in the completion of nursing care tasks and documentation following the introduction of bedside handovers. Specifically, they reported higher rates of allergy alert band usage (from 83.3% to 95.4%), medications administered as prescribed (from 81.1% to 97.3%), and proper labeling on medication charts (from 78.7% to 96.8%). Moreover, substantial improvements were observed in nursing documentation, including admission forms, Braden tool assessments at various time points, and intravenous cannula documentation.

### 3.6. Patient-Related Outcomes

Bedside handovers have a significant impact on various aspects of patient care. The available evidence points to clear benefits in terms of patient satisfaction, involvement, anxiety reduction, and perceived safety. In Table 3, the key quantitative outcomes are summarized. However, concerns remain regarding privacy and consistency of implementation. Below, we reorganize the patient-related outcomes according to the main key dimensions identified.

#### 3.6.1. Patient Satisfaction

Numerous studies [30, 32, 35] demonstrate that bedside handovers enhance patient satisfaction by fostering transparency and involving patients in care decisions. Cairns et al. [30] reported significant increases in satisfaction scores following bedside shift reports, including improved ratings for “Nurses kept you informed” rising from 73.8 to 88.9, and “Staff included you in decisions regarding treatment” increasing from 69.7 to 79.9, as measured via the Press Ganey Satisfaction survey. Similar improvements were observed in other studies, where patients expressed appreciation for better communication and the opportunity to participate in care planning [32, 35, 38].

Qualitative findings reinforce this perspective. Patients described feeling reassured and valued when healthcare professionals demonstrated knowledge and openness during handovers [29, 33]. Some participants highlighted that being informed helped them feel “protected” and gave them a sense of “security” during their recovery [33]. Others noted that hearing staff communicate clearly at the bedside made them feel included and respected [36].

While most patients appreciated the inclusive approach, preferences varied. Some individuals preferred to listen passively, while others felt their voices were finally being heard [27, 29, 33]. This variability underlines the importance of tailoring the handover process to individual patient needs.

#### 3.6.2. Patient's Involvement

Patient involvement in bedside handovers has received mixed reactions across studies [28, 29, 33, 39]. Many patients appreciated the opportunity to listen and contribute to discussions about their care, feeling more connected and reassured. For instance, one patient shared, “*It's great to be able to know who is looking after you… before you used to ring the bell and it'd be anyone's guess*” [28]. Others valued the chance to ask questions or verify information, with one noting, “*It is interesting to hear what they are doing for you and what they are planning… I feel like now my thoughts and opinions on my care count*” [28].

However, some patients preferred to remain passive observers, finding comfort in simply overhearing the process [29]. Others felt excluded when nurses primarily communicated with each other. One participant commented, “*They don't tell you anything; I get that they have to talk to one another, but they should involve the patient if there's something concerning him*” [33].

#### 3.6.3. Patient Safety and Privacy

Patient safety was another domain positively influenced by bedside handovers. By promoting accurate information exchange and allowing patients to verify details, this practice helps prevent errors and fosters a collaborative care environment. For example, Bruton et al. [29] noted that patients felt safer knowing all staff members were well-informed about their condition. Some even viewed the process as a “safety net” allowing them to detect and address discrepancies in care.

Similarly, Lupieri et al. [33] highlighted that patients derived reassurance from seeing nurses discuss and resolve issues in real time. In some cases, the emotional security provided by bedside communication helped patients cope with pain and stress: “*It provides you a sense of safety*… *when someone tells you ‘everything went OK, I fixed it,' you can stand the pain*” [33]. Lu et al. [36] confirmed that patients believed their presence during handovers could reduce the likelihood of mistakes and enhance quality of care.

Nevertheless, safety is also tied to privacy. Many patients raised concerns about confidentiality in shared rooms or when discussing sensitive topics such as mental health or sexual health. While some were unconcerned about privacy breaches, others emphasized the need for discretion, especially in the presence of visitors or when topics of a highly personal nature were involved [33, 36, 39].

Family presence was another variable. Some patients appreciated having relatives present to support their understanding, while others preferred the discussion remain private between staff and patient: “*They should use their own discretion and ask visitors to leave before discussing handover* [39].”

#### 3.6.4. Patient Anxiety

Bedside handovers appear to reduce patient anxiety by promoting consistent, real-time communication and enhancing patients' understanding of their care. Baldwin and Spears [27] reported that patients felt “anxiety-free” after being included in bedside reports. This sense of calm was linked to knowing what was happening and who was in charge of their care.

However, this benefit may be undermined by unclear or overly technical language. Kerr et al. [39] and Lu et al. [36] noted that the use of medical jargon occasionally confused patients, potentially generating new concerns. Patients suggested that clearer explanations and consistent formats could further mitigate anxiety and enhance understanding. Thus, while the inclusion of patients in bedside handovers generally alleviates stress, thoughtful communication practices are essential to maintain this benefit.

#### 3.6.5. Personalized Care, Quality of Care, and Empowerment

Bedside handovers have also been linked to perceptions of personalized and high-quality care. Kullberg and Malfait [15, 32] utilized the Individualized Care Scale (ICS) as a secondary outcome measure. The ICS is a two-part instrument (ICS‐A/ICS‐B) comprised of 17 items each: The ICS‐A measures patients' ratings of how nursing care supports individuality, while the ICS‐B assesses how individuality is perceived in the care received. Both studies showed that patients generally rated their care as more individualized than the explicit nursing activities provided. In Malfait's study, the intervention group scored higher, with mean scores rising from 3.826 to 3.910, compared to the control group's increase from 3.599 to 3.791. Although improvements were noted in both ICS-A and ICS-B, these were not statistically significant (ICS-A: *p*=0.427; ICS-B: *p*=0.710). Kullberg's research [32] also reflected these trends, with patients rating part B of the ICS higher, indicating positive perceptions of care, though the “Personal life situation” dimension received the lowest scores, highlighting an area for improvement.

Qualitative research further emphasizes the empowering nature of bedside handovers. Patients appreciated the transparency and the opportunity to contribute to discussions about their care [36]. One patient remarked, “*Having them discuss your case in front of you relieves any doubt you are hearing everything*” [27]. However, preferences varied, with some patients preferring a more passive role. Others worried that their involvement might distract nurses or cause logistical challenges, such as being woken during handovers.

## 4. Discussion

This is the first systematic review that investigates the impact of bedside handovers on clinical and organizational outcomes within hospital settings. A total of 13 studies, both qualitative and quantitative, were analyzed. The primary objective was to evaluate how the introduction of nursing handovers at the patient's bedside—an approach that actively involves patients during shift changes—can improve safety, satisfaction, and organizational efficiency in hospital environments.

The results underscore the significant role of bedside handovers in enhancing continuity of care by improving the accuracy and timeliness of information exchange, increasing patient involvement through direct participation and opportunities to ask questions, and optimizing organizational processes by reducing handover duration, minimizing nursing overtime, and decreasing call bell usage.

However, despite these advantages, the implementation of bedside handovers remains variable in current clinical practice. Our findings highlight that the success of this approach depends not only on its structure but also on contextual factors such as staff training, unit culture, and available resources. Existing literature supports this view, noting that standardization tools like ISBARR can enhance the clarity and consistency of handovers [34]. Moreover, although some patients value active participation, nurses must balance this with time constraints and the need to maintain focus, especially in shared environments. Effective integration of bedside handover into routine practice requires alignment with institutional privacy policies and tailored communication strategies that respect individual patient preferences.

There is consistent evidence supporting the positive effect of bedside handovers on patients' perceptions of safety and overall satisfaction. Bressan et al. [20], in their umbrella review, found that patients are supportive of bedside shift reports as a right, as an opportunity to be involved, and of being in the center of the nursing care process [20] and our findings confirm this, with both qualitative and quantitative studies supporting this hypothesis [29, 30, 32, 33, 35, 38].

However, researchers and practitioners have raised several concerns regarding the implementation of bedside handovers, which have impeded its widespread adoption [35]. These concerns include, among others, the potential increase in report time, the possibility of patients or families monopolizing the conversation [41], and the risk of breaching patient privacy [18, 36, 42]. However, evidence from the studies reviewed in this paper suggests that these concerns may be less significant than initially perceived. Research indicates that while bedside handovers may slightly extend handover time, they contribute to an overall reduction in total care time, as reflected in decreased nursing staff overtime. Furthermore, studies show that patients are not always concerned about privacy breaches during bedside handovers and do not necessarily wish to actively participate or dominate the discussion.

Although Malfait et al. [34] observed a slightly increased duration of individual bedside handovers (from 75–180 to 83–204 s), they also reported a significant reduction in total nursing shift overtime. This suggests that more effective communication and planning during bedside handover can streamline subsequent care activities. Similarly, Cairns et al. [30] reported a cumulative reduction of 913 min of overtime during their study period—equivalent to approximately 10 min per day or 61 h annually—indicating that any minor increase in handover time may be offset by greater overall time efficiency during the shift.

The familiarity that nurses develop with patients during bedside handovers, including a thorough review of their overall condition and medical devices, allows for greater efficiency and time savings during shifts. Notably, both Cairns et al. [30] and Bradley and Mott [28] reported that bedside handovers led to a decrease in patient call frequency, suggesting that more engaged and informed patients are less likely to request assistance, thus reducing interruptions in nursing workflows. This observation is particularly relevant in the current context of a global shortage of healthcare personnel [43], where improving work efficiency is critical to compensating for staffing shortages. Moreover, Kerr et al. [37] confirmed that bedside handovers improve the completion of nursing care tasks and documentation, highlighting the link between structured communication and adherence to clinical protocols.

Although there are concerns that patients might monopolize the conversation, our review shows that their preferences are diverse. Some patients prefer a more passive role, observing the process and gaining insights into their care [29], while others express concerns that their involvement could divert nurses' attention or create logistical challenges [28]. On the other hand, some patients feel that bedside handovers provide them with a sense of reassurance, ensuring transparency in communication [28]. The reviewed studies indicate that there is no substantial evidence suggesting that patient participation prolongs or delays the handover process [27, 29].

Privacy concerns remain a significant consideration, with varying perspectives among patients. Some consider privacy a crucial factor, emphasizing the importance of discretion in shared hospital environments and the need for confidentiality in handling medical information. Conversely, others view privacy as a secondary concern, believing that sharing information in a hospital setting is a natural aspect of their stay [33]. The issue of privacy also extends to the presence of caregivers and family members during handovers. Most patients prefer that visitors and family members leave the room during the process to maintain confidentiality and ensure a more focused exchange of information. In such cases, patients expect nurses to take the initiative in asking visitors to leave the room before proceeding with the handover [39].

### 4.1. Limitations

The main limitation of this review is the substantial heterogeneity among the studies analyzed, due to differences in study design, sample sizes, characteristics of the interventions (e.g., duration and frequency of monitoring), participant characteristics, and assessment tools. The diversity of the operational units considered may affect the generalizability of the results. Additionally, the measurement of organizational outcomes presents challenges. Variables such as reductions in overtime or improvements in compliance may be influenced by external factors that were not controlled for in the included studies. As a result, the actual impact of bedside handovers on hospital efficiency may be difficult to quantify accurately. Finally, there is significant variation in bedside handover models across different hospitals and units, with differences in structure, communication techniques, and patient involvement levels. This variability complicates direct comparisons and limits the ability to draw definitive conclusions about the best practices for implementing bedside handovers effectively.

## 5. Conclusion

In conclusion, the implementation of bedside handovers presents both opportunities and challenges. While concerns regarding increased time, patient involvement, and privacy remain, the overall benefits—such as improved communication, increased patient engagement, and enhanced efficiency—outweigh the potential drawbacks. Bedside handovers contribute to a more patient-centered approach, fostering better relationships between patients and healthcare providers and optimizing organizational outcomes. Healthcare institutions should focus on addressing barriers to implementation through targeted training, clear guidelines, and ensuring a patient-centered approach that respects individual preferences and privacy needs. Future research should continue to explore strategies for enhancing the effectiveness and acceptance of bedside handovers across various healthcare settings.

## Figures and Tables

**Table 1:** Summary of the characteristics of included studies.

Authors, year	Study design	Country, setting	Population	Aim	Intervention or measurement	Main findings	Quality of study
Bradley S. et al., 2013	Mixed methods study	Australia, acute ward	Patients (*n* = 9) Nurses (*n* = 42)	To explore the process and outcome of the introduction of bedside handover	Staff interview Staff survey (19 items) Patient interview	Patient involvement in the handover process significantly increased, with scores rising from 2.8–3.4 before the intervention to 4.6–5.8 afterward. Perceptions of exclusion in information transfer also decreased, with scores dropping from 4.04–5.09 preimplementation to 2.4–4.2 postimplementation. Interviews highlighted three key themes: social interaction, nurse involvement, and patient care. Patients valued the nurse-to-nurse bedside handover for enhancing social interaction, allowing them to meet their nurses, and providing a greater sense of involvement in their care, with more information shared, including test results	Moderate

Kullberg A. et al., 2018	Cross-sectional	Sweden, oncology	Patients (*n* = 90)	Assess patient's satisfaction 2 years after implementation of bedside handover	EORTC INPATSAT 32 questionnaire, ICS (individualized care scale)	Statistically significant improvements in patient satisfaction were observed in the subscales “Exchange of information between caregivers” (*p* < 0.001) and “nurses' information provision” (*p*=0.028) after implementing person-centered handover. The highest scores for individualized care perceptions were in the ICS-A subscale “clinical situation” (3.85) and ICS-B “decisional control” (3.91).	Moderate

Scheidenhelm S. et al., 2017	Before—after	USA, community hospital	*Before:* Patients (*n* = 290) nurses (*n* = 132) *After:* Patients (*n* = 289) nurses (*n* = 202 after 1 month, *n* = 147 after 3 months)	Investigate patient satisfaction and nurse compliance before and after the implementation of bedside handover	HCAHPS survey (29 items) and direct nurses' observation	Intervention increases nurse compliance with bedside handover—from 55.9 to 90.6 in the obstetric unit, from 12.3 to 84 in medical/surgical unit. Satisfaction in general improves after the intervention but it was not statistically significant.	Moderate

Bruton J. et al., 2016	Qualitative	UK, acute wards	Patients (*n* = 8), nurses (*n* = 10), nurse student (*n* = 1) healthcare assistants (*n* = 3), doctor (*n* = 1), physiotherapist (*n* = 1)	Understand the purpose, impact, and experience of bedside handover from the patients and the staff perspective.	Interview	The bedside handover facilitated clinical information exchange, though without a standardized format. Benefits included introducing the incoming nurse, visually assessing the patient, and ensuring continuity of care. Challenges included speaking over patients, breaches of confidentiality, and interruptions. Patients felt reassured when nurses were well-informed but insecure when uninformed. Some wanted more involvement, while others preferred passive listening. Clear communication and updates were valued, though rigid routines sometimes hindered the experience.	High

Kerr D. et al., 2014	Qualitative	Australia, emergency department	Patients (*n* = 30)	Explore the perspective of patients about bedside handover in emergency department	Thematic analysis	Two main themes emerged: (1) Patients feel that participating in bedside handovers enhances individualized care, allowing them to clarify discrepancies and contribute additional information. It reassures them about nurses' competence and care continuity. (2) Maintaining privacy and confidentiality is crucial. Patients prefer handovers in the emergency department cubicle to protect their information and expect discretion with sensitive or new details.	High

Lupieri G. et al., 2015	Qualitative	Italy, cardiothoracic intensive care unit	Patients (*n* = 14)	Describe the experiences of patients experiencing nursing bedside handover	Interview	Four main themes emerged: (1) Discovering a new nursing identity: Patients gained a positive view of nursing, feeling reassured that their condition was well-communicated, despite some confusion between nurse and physician roles. (2) Partial engagement in bedside handover: Patients appreciated bedside handovers but wanted more involvement, feeling sidelined when nurses focused on each other. Bad news during handovers increased anxiety. (3) The paradox of confidentiality: While privacy was maintained through soft speech, most patients prioritized the benefits of hearing the handover over confidentiality concerns. (4) Having control over the situation: Patients felt reassured and empowered by participating in handovers, allowing them to verify the accuracy of shared information.	High

Malfait S. et al., 2018	Before-after	Belgium, geriatric, medical rehab, and surgical wards	*Before*: Nurse (*n* = 105), handover observation (*n* = 40) *After*: Handover observation (*n* = 50)	To explore the effects of bedside handover on handover duration.	*Before:* Nurse interview, observations of the handover, timing duration of shift handover *After*: Bedside handover observation, timing the duration	The average bedside handover time was 146 s (range: 83–204 s). The introduction of a decentralized handover model and ISBARR resulted in varied time outcomes across wards. In one group, decentralization reduced total handover time by 24%, but increased time per patient. In another group with both decentralization and ISBARR, total handover time decreased by 54%–68%, with time per patient stable or slightly increased. A third group saw a 15%–45% reduction in total handover time but a 60% increase in time per patient. In a fourth group with only ISBARR, both total time and time per patient decreased by 27%. The fifth group, already using both methods, saw no significant change (−4%–26%).	Moderate

Baldwin K. et al., 2019	Mix method study	USA, medical/surgical wards	*Intervention group*: Patient (*n* = 35) *Control group*: Patient (*n* = 38)	Measure patient's anxiety on admission to the hospital and evaluate the patient's experience and opinion about the nursing bedside report.	Beck anxiety inventory	There was no significant difference in baseline anxiety scores between the intervention and control groups (*p*=0.6363). Anxiety levels generally decreased in participants of the nursing bedside report, especially as they began to feel better. In contrast, anxiety levels in the control group remained more stable throughout hospitalization.	Moderate

Malfait S. et al., 2019	Before-after	Belgium, geriatric, medical rehab, and surgical wards	*Intervention group*: Patient (*n* = 524), nurse (*n* = 91) *Control group*: Patient (*n* = 275), nurse (*n* = 74)	Investigate the effectiveness of bedside handover.	PAM13 QPP ICS-MOAQ CPSET ICS-self-developed 10-point Likert scale for nurse's perceptions of work interruptions.	No significant changes were observed in patient outcomes, including individualized care, quality of care, or patient activation, in either group, except for an improvement in physical-technical conditions in the control group (*p* < 0.001). Nurses in the intervention group showed stable individualized care, while it significantly declined in the control group (*p*=0.015). Patient participation increased significantly in the intervention group (*p*=0.001), with no change in the control group. Work interruptions were significantly reduced in the intervention group (*p*=0.016), with no changes in the control group. Some aspects of care, including individualized care subscales, declined in the control group (*p*=0.008). Overall, the intervention group showed stable or improved outcomes, while the control group saw declines.	High

Maxson P. et al., 2012	Before-after	USA, surgical ward	Patients (*n* = 30), nurse (*n* = 15)	Determinate if bedside handover increases patient satisfaction and patient perception of teamwork and increases staff satisfaction	An original survey for patients and an original survey for staff.	The mean scores of the patient survey (1 = best, 5 = worst) ranged from 1.5 to 2 before the practice change and improved to a mean of 1 after the change. A significant improvement was observed in the question regarding whether patients were informed about their daily care plan (*p*=0.02).	Moderate

Cairns L. et al., 2013	Before-after	USA, trauma unit	N/A	Evaluate the effects of redesign of shift handoff report measured by the amount of end of shift overtime, frequency of call light usage during change of shift. and patients/nurses' perception	- 7 questions survey - Press Ganey Patient Satisfaction survey - Measure of overtime	End of shift overtime minutes decreased from 6194 min before to 5281 after implementation, call light usage during change of shift decreased by 33%, the mean satisfaction score increased from 73.8 before to 88.9 after implementation, more nurses agree after implementation that the report was concise and contained information pertinent to patients' conditions and that the report was more consistent, and 50% of the nursing staff reported that teamwork and accountability improved	Moderate

Kerr D. et al., 2013	Before-after	Australia, acute medical, acute surgical, and maternity wards	Before: Handover observation (*n* = 15), medical records (*n* = 381) After: Handover observation (*n* = 15) medical records (*n* = 373)	To examine whether the introduction of bedside handover improves the completion of nursing care tasks and documentation	- Handover duration - 3 nursing care tasks (allergy alert band, medication administration, and medication chart labeling) - 7 documentation items (admission form, Braden scale at admission, 2 days, and 1 week, pressure ulcer prevention measures, and intravenous cannula assessment)	Significant improvements were observed in the completion of nursing care tasks (allergy alert band: 83.3% vs. 95.4%; medications administered as prescribed: 81.1% vs. 97.3%; identification labels on the medication chart: 78.7% vs. 96.8%). Documentation completion also improved (admission form: 78.2% vs. 92.3%; Braden tool on admission: 73.6% vs. 91.8%; Braden tool at 2 days: 70% vs. 84.8%; Braden tool at 1 week: 51.5% vs. 100%; intravenous cannula assessment: 53.3% vs. 81.2%). No significant decrease in handover duration was observed.	High

Lu S. et al., 2014	Qualitative	Australia, acute medical ward, acute surgical ward, maternity	Patients (*n* = 30)	Describe the experiences of patients experiencing nursing bedside handover	Interview	Four themes emerged: (1) Personalized approach: Patients reported feeling more actively involved in their care, and bedside handovers enabled nurses to develop a deeper understanding of the patient's individual needs and experiences. (2) Safety and empowerment: Patients expressed a desire to be informed about their health status. Bedside handovers facilitated this by allowing them to follow the clinical discourse, thereby enhancing their sense of control and enabling them to detect or prevent potential errors in their care. (3) Privacy: While most patients felt comfortable discussing their condition, concerns were raised regarding the discussion of sensitive or personal issues in shared spaces, particularly in the presence of other patients with unstable mental health conditions. (4) Comprehension: Patients emphasized the importance of using clear and accessible language during handovers to fully understand their medical situation and ongoing care.	High

*Note:* EORTC INPATSAT 32: European Organisation for Research and Treatment of Cancer In-Patient Satisfaction with Care Questionnaire; PAM13: 13‐item patient activation measurement; QPP: Quality from the Patient's Perspective questionnaire; ICS‐patient: individualized care scale for patients; ICS‐A: Patient explores the patient's perceptions on how nurses support patient's individuality through nursing activities; ICS‐B: Patient explores the degree to which the patient perceives his/her care as an individual; CPSET: Care Process Evaluation Tool; ICS: Nurse individualized care scale for nurses.

Abbreviations: HCAHPS, Hospital Consumer Assessment of Healthcare Providers and Systems; MOAQ, Michigan Organizational Assessment Questionnaire.

**Table 2:** Quantitative organizational outcomes: nurse compliance, handover duration and overtime, overtime and call light activation, nursing case tasks, and documentation completion.

**Authors, year**	**Outcome**	**Measurement**		**Intervention duration**		
**Nurse compliance**				**T0**	**1 month**	**2 months**

Scheidenhelm S. et al., 2017	Nurse compliance with bedside report	Direct observations in obstetrics unit	%	55.9	83.6 (after 1 month)	90.6 (after 3 months)
		Direct observation in medical/surgical unit		12.3	85 (after 1 month)	84 (after 3 months)

**Handover duration and overtime**				**T0**	**3 months**	

Malfait S. et al., 2018	Handover duration	Average time per patient used for handover	Sec.	75–180	83–204	
	End-of-shift overtime	% of wards reporting overtime due to handover	%	58.3	8.3	

				**T0**	**12 months**	**p**

Kerr D., et al., 2013	Handover duration	Average handover time	Min.	33.5	30.6	0.880

**Overtime and call light activation**				**T0**	**3 months**	

Cairns L. et al., 2013	End-of-shift overtime		Min. (h)	6194 (103)	5281 (88)	% Of change: 15%
	Call light activation	Call light usage	n.	1591	1075	% Of change: 33%

**Nursing care tasks completion**				**T0**	**12 months**	**p**

Kerr D., et al., 2013	Allergy alert band	Number allergic patients wearing allergy alert band	*n* (%)	80/96 (83.3)	124/130 (95.4)	0.003
	Medication administered	Number of medications administered as prescribed		308/380 (81.1)	362/372	< 0.001
	Medication chart labeling	Number of indication labels on each side of the medication chart		300/381 (78.7)	361/373 (96.8)	< 0.001

**Nursing documentation completion**				**T0**	**12 months**	**p**

Kerr D., et al., 2013	Nursing documentation completion	Number of admission form	*n* (%)	154/197 (78.2)	192/208 (92.3)	< 0.001
		Number of Braden tool completion on admission		89/121 (73.6)	191/208 (91.8)	< 0.001
		Number of Braden tool completion 2 days after admission		56/80 (70)	112/132 (84.8)	< 0.010
		Number of Braden tool completion 1 week after admission		17/33 (51.5)	38/38 (100)	< 0.001
		Number of preventive measures for pressure ulcer on admission		73/108 (67.6)	130/208 (62.5)	0.370
		Number of preventive measures for pressure ulcer 2 days after admission		46/76 (60.5)	90/131 (68.7)	0.232
		Number of intravenous cannula assessment		48/90 (53.3)	65/80 (81.2)	< 0.001

**Table 3:** Quantitative patient outcomes: patient satisfaction, patient's involvement, and patient safety.

**Authors, year**	**Measurement**	**Outcomes**		**Intervention duration**		
**Patient satisfaction**				**Previous study**	**Current study**	

Kullberg A. et al., 2018	EORTC INPATSAT 32 questionnaire 0-100 SCALE	Exchange of information	Mean (SD)	62	77	
	ICS A 5‐point Likert scale 1 = “strongly disagree” 5 = “strongly agree”	Support of individuality			3.46 (3.19–3.73)	
		Clinical situation			3.71 (3.46–3.96)	
		Personal life situation			3.03 (3.21–3.73)	
		Decisional control			3.47 (3.21–3.73)	
	ICS B 5‐point Likert scale 1 = “strongly disagree” 5 = “strongly agree”	Perception of individuality			3.69 (3.42–3.96)	
		Clinical situation			3.85 (3.6–4.1)	
		Personal life situation			3.07 (2.77–3.37)	
		Decisional control			3.91 (3.66–4.16)	

				**T0**	**5 months**	

Scheidenhelm S. et al., 2017	HACHPS survey in obstetrics unit	“Nurses kept you informed”	Mean (SD)	96.56 (10.48)	96.36 (10.44)	
		“Staff included you in decision regarding treatment”		94.26 (13.95)	95.51 (9.32)	
		“Communication with nurses”		90.60	94.60	
		“Nurses explained in a way you understand”		97.80 (7.21)	97.55 (8.34)	
	HCAHPS survey in medical/surgical unit	“Nurses kept you informed”		89.95 (15.99)	92.74 (12.84)	
		“Staff included you in decision regarding treatment”		89.11 (16.12)	91.16 (12.88)	
		“Communication with nurses”		79.60	86.80	
		“Nurses explained in a way you understand”		92.22 (14.79)	94.30 (11.54)	

**Patient's involvement**						

Bradley	7‐point Likert scale	“I believe that the patient is involved in the handover process that occurs between shifts”	Mean (SD)			
			Site 1	2.867 (0.374)	5.486 (0.402)	
			Site 2	3.000 (0.402)	5.583 (0.418)	
			Site 3	3.455 (0.437)	4.667 (0.483)	

**Patient safety**						
				**T0**	**1 month**	

Maxson P. et al., 2012	Survey, 1 = best 5 = worst	“I was informed of my plan of care for the day”		2 (1–2.25)	1 (1–2) *p* 0.02	
		“There was open communication between members of the healthcare team about my plan of care”		2 (1–2)	1 (1–2) *p* 0.06	
		“I was satisfied with the amount of input I was able to give about my plan of care”		1.5 (1–2)	1 (1–2) *p* 0.37	
		“My care providers worked together as a team”		1.5 (1–2)	1 (1–2) *p* 0.14	
		“The report given between care providers was given in a professional and confidential manner”		2 (1–2)	1 (1–2) *p* 0.1	

				**T0**	**3 months**	**9 months**

Malfait S. et al., 2019	PAM13	Patient activation measurement-intervention	Mean (SEM) range 1–5	3.094 (0.038)	3.101 (0.038)	3.210 (0.039)
		Patient activation measurement-control		3.011 (0.052)	3.030 (0.048)	3.314 (0.058)
	ICS-patient	Individualized care scale for patients-intervention		3.826 (0.063)	3.787 (0.064)	3.910 (0.064)
		Individualized care scale for patients-control		3.599 (0.088)	3.724 (0.082)	3.791 (0.089)
	ICS-A-patient	How nurses support patient's individuality (patient's perceptions)—Intervention		3.690 (0.074)	3.612 (0.075)	3.767 (0.077)
		How nurses support patient's individuality (patient's perceptions) control		3.448 (0.103)	3.582 (0.096)	3.609 (0.114)
	ICS- B-patient	How patient perceives his/her care as an individual-intervention		3.964 (0.066)	3.969 (0.067)	4.069 (0.069)
		How patient perceives his/her care as an individual-control		3.753 (0.092)	3.8557 (0.085)	3.976 (0.101)
	QPP	Patient's perspective questionnaire-intervention		3.634 (0.047)	3.704 (0.048)	3.682 (0.048)
		Patient's perspective questionnaire-control		3.466 (0.063)	3.536 (0.059)	3.685 (0.064)

*Note:* EORTC INPATSAT 32: European Organisation for Research and Treatment of Cancer In-Patient Satisfaction with Care Questionnaire, ICS- Nurse individualized care scale for nurses ICS‐A‐Patient explores the patient's perceptions on how nurses support patient's individuality through nursing activities. ICS‐B Patient explores the degree to which the patient perceives his/her care as an individual. PAM13 13‐item patient activation measurement QPP: Quality from the Patient's Perspective questionnaire.

## Data Availability

The data that support the findings of this study are available in the supporting information of this article.

## Supporting Information

Supporting Table 1. Criteria for study selection according to the PICOS model (Population, Intervention, Comparison, Results, Study design) and criteria for data extraction and management.

Supporting Table 2. Search strategies.

Supporting Table 3. Methodological quality of the cross-sectional studies.

Supporting Table 4. Methodological quality of the qualitative research.

Supporting Table 5. Methodological quality of the quasi-experimental studies.
