# Supplementary material for: Impact of Bedside Handover on Patient Perceptions and Hospital Organizational Outcomes: A Systematic Review
Source: J Nurs Manag. 2025 Jul 21;2025:3803491. doi: 10.1155/jonm/3803491 (PMC12303637; doi:10.1155/jonm/3803491)
Supplement: Supporting Information — Additional supporting information can be found online in the Supporting Information section. [file 3803491.f1.docx]

**Impact of Bedside Handover on Patient perceptions and Hospital Organizational Outcomes: A Systematic Review**

**Supplementary Material**

**CONTENTS**

**[Supplemental Table 1](https://docs.google.com/document/d/1jIT6kJlrYvAy1ndzWN4paFUVivBFxQrM/edit#heading=h.gjdgxs)**[. Criteria for study selection according to the PICOS model (Population, Intervention, Comparison, Results, Study design) and criteria for data extraction and management](https://docs.google.com/document/d/1jIT6kJlrYvAy1ndzWN4paFUVivBFxQrM/edit#heading=h.gjdgxs)

**Supplemental Table 2**. Search strategies

**[Supplemental Table 3](https://docs.google.com/document/d/1jIT6kJlrYvAy1ndzWN4paFUVivBFxQrM/edit#heading=h.2et92p0)**[.](https://docs.google.com/document/d/1jIT6kJlrYvAy1ndzWN4paFUVivBFxQrM/edit#heading=h.2et92p0) Methodological quality of the Cross-Sectional Studies

**[Supplemental Table 4.](https://docs.google.com/document/d/1jIT6kJlrYvAy1ndzWN4paFUVivBFxQrM/edit#heading=h.2et92p0)** [Methodological quality of the qualitative research.](https://docs.google.com/document/d/1jIT6kJlrYvAy1ndzWN4paFUVivBFxQrM/edit#heading=h.2et92p0)

**[Supplemental Table 5](https://docs.google.com/document/d/1jIT6kJlrYvAy1ndzWN4paFUVivBFxQrM/edit#heading=h.3dy6vkm)**[. Methodological quality of the Quasi experimental studies](https://docs.google.com/document/d/1jIT6kJlrYvAy1ndzWN4paFUVivBFxQrM/edit#heading=h.3dy6vkm)

**Supplemental Table 1. Criteria for study selection according to the PICOS model (Population, Intervention, Comparison, Results, Study design) and criteria for data extraction and management.**

| **Study selection** |  |
| --- | --- |
| **Population** | Hospitalized adult patients |
| **Intervention** | Bedside Handover |
| **Comparator** | Other type of shift handover |
| **Outcomes** | I)Staff compliance, II) reduction in handover duration III) overtime hours reduction IV)cost savings V)call light usage VI)patient’s satisfaction VII)patient’s safety VIII)quality of care IX)patient’s anxiety X)patient’s involvement. |
| **Study design** | I)Randomized clinical trials (RCTs) II) observational studies (cohort, case-control, cross-sectional studies) III) exploratory and quantitative studies IV) qualitative study |
| **Inclusion criteria** | I) study population: humans, adults; II) study type: randomized controlled trials, observational studies (cohort, case-control, cross-sectional studies), exploratory studies, mix of qualitative and quantitative studies; III) review articles were excluded, but their reference lists were screened to identify potential eligible studies; IV) only published full papers were included, whereas abstracts only were not included; V) data on nursing nutritional intervention/counseling, relation with LOS, mortality, number of infections, nutritional intake and anthropometrics; VI) publication date: last 20 years (2004-2024). |
| **Exclusion criteria** | I) full paper not available; II) study not yet published; III) studies not reporting data on bedside handover; IV) review; V) study protocols; VI) case reports or case series; VII) articles published in languages other than English; VIII) non-human studies; IX) non-hospitalized patients, X) paediatric patients. |
| **Characteristics for paper analysis** | I) reference details: authorship(s); published or unpublished; year of publication; period in which the study was conducted; other relevant cited papers; II) study characteristics: study design, topic, treatment period, follow up duration, region; III) population characteristics: number of participants, data on age, hospital setting; IV) methodology: assessment of the type of nursing nutritional intervention; V) main results: organizational outcome, patients satisfaction, patients safety. |

**Supplemental Table 2: Search strategies**

| **(P)**ICO | P**(I)**CO | PIC**(O)** | PICO**(S)** |
| --- | --- | --- | --- |
|  |  |  |  |
| ("hospitalized" OR "inpatient" OR "inpatients" OR "hospitalization" OR "hospitals" OR "hospitalized patients" OR "patients" OR “client” OR “consumer*”"nursing" OR "nursing care" OR "nurse*" OR "nursing staff" OR "healthcare workers" OR "healthcare providers") | ( "handover" OR "handoff" OR "nursing handover" OR "nursing handoff" OR "patient handoff" OR "patient handover" OR "patient transfer" OR "sign out" OR "inter shift" OR "shift report" OR "change of shift" OR "shift change" OR "service change" OR "transition of care" OR "bedside report" OR "bedside handover" OR "patient round" OR "shift handoff") | ("patient outcomes" OR "patient satisfaction" OR "quality of care" OR “quality improvement” OR "patient experience" OR "continuity of care" OR "safety" OR "error reduction" OR "therapeutic relationship" OR “time” OR "communication" OR "patient-centered care" OR "clinical outcomes" OR "patient participation" OR "nurse-patient relations"  OR “Patient Safety” OR “continuity of patient care”) | ("randomized controlled trial" OR "clinical trial" OR "observational study" OR "comparative study" OR "qualitative study" OR "cohort study" OR "cross-sectional study" OR "case study" OR "prospective study" OR "retrospective study" OR "mixed-methods study" OR "case-control study") |

**Supplemental Table 3.** **Methodological quality of the Cross Sectional Studies**

| **Criterion** | **Kullberg et al, 2018** |
| --- | --- |
| **1.Were the criteria for inclusion in the sample clearly defined?** | - |
| **2. Were the study subjects and the setting described in detail?** | + |
| **3.Was the exposure measured in a valid and reliable way?** | + |
| **4.Were objective, standard criteria used for measurement of the condition?** | + |
| **5.Were confounding factors identified?** | + |
| **6.Were strategies to deal with confounding factors stated?** | - |
| **7.Were the outcomes measured in a valid and reliable way?** | + |
| **8.Was appropriate statistical analysis used?** | + |
| **Quality of the study** | MODERATE QUALITY |

+=Yes; -=No; ?=“Unclear”; N/A: not applicable

**Supplemental Table 4**. **Methodological quality of the qualitative research**

| **Criterion** | **Bruton et al, 2016** | **Kerr et al, 2014** | **Lupieri et al, 2015** | **Bradley, S. & Mott, S., 2013** | **Chaboyer, W., et al. 2008** | **Baldwin K. et al. 2019** | **Lu, S. et al., 2014** |
| --- | --- | --- | --- | --- | --- | --- | --- |
| **1. Is there congruity between the stated philosophical perspective and the research methodology?** | **+** | **+** | **+** | **+** | **-** | **+** | **+** |
| **2. Is there congruity between the research methodology and the research question or objectives?** | **+** | **+** | **+** | **+** | **-** | **+** | **+** |
| **3. Is there congruity between the research methodology and the methods used to collect data?** | **+** | **+** | **+** | **+** | **-** | **+** | **+** |
| **4. Is there congruity between the research methodology and the representation and analysis of data?** | **+** | **+** | **+** | **+** | **-** | **+** | **+** |
| **5. Is there congruity between the research methodology and the interpretation of results?** | **+** | **+** | **+** | **+** | **-** | **+** | **+** |
| **6. Is there a statement locating the researcher culturally or theoretically?** | **-** | **-** | **+** | **+** | **-** | **-** | **-** |
| **7. Is the influence of the researcher on the research, and vice- versa, addressed?** | **+** | **+** | **+** | **+** | **-** | **-** | **+** |
| **8. Are participants, and their voices, adequately represented?** | + | + | + | + | - | + | + |
| **9. Is the research ethical according to current criteria or, for recent studies, and is there evidence of ethical approval by an appropriate body?** | + | + | + | + | - | - | + |
| **10. Do the conclusions drawn in the research report flow from the analysis, or interpretation, of the data?** | + | + | + | + | - | + | + |
| **Quality of the study** | HIGH QUALITY | HIGH QUALITY | HIGH QUALITY | HIGH QUALITY | LOW QUALITY | MODERATE QUALITY | HIGH QUALITY |

**Supplemental Table 5.** **Methodological quality of the Quasi experimental studies**

| **Criterion** | **Scheidenhelm S., 2017** | **Bradley, S. & Mott, S., 2013** | **Malfait S. et al. 2018** | **Maxson P. et al, 2012** | **Malfait S. et al. 2019** | **Baldwin K. et al. 2019** | **Cairns, L.L., 2013** | **Kerr D. et al, 2013** |
| --- | --- | --- | --- | --- | --- | --- | --- | --- |
| **1.Is it clear in the study what is the “cause” and what is the “effect” (i.e. there is no confusion about which variable comes first)?** | + | + | + | + | + | + | + | + |
| **2. Was there a control group?** | - | - | - | - | - | - | - | + |
| **3. Were participants included in any comparisons similar?** | + | + | - | + | + | + | + | + |
| **4. Were the participants included in any comparisons receiving similar treatment/care, other than the exposure or intervention of interest?** | - | + | + | + | + | + | + | + |
| **5. Were there multiple measurements of the outcome, both pre and post the intervention/exposure?** | + | - | + | + | + | - | + | + |
| **6. Were the outcomes of participants included in any comparisons measured in the same way?** | + | + | - | + | + | + | + | + |
| **7.Were outcomes measured in a reliable way?** | + | + | + | + | + | - | + | + |
| **8. Was follow-up complete and if not, were differences between groups in terms of their follow-up adequately described and analyzed?** | N/A | N/A | + | N/A | + | + | N/A | + |
| **9. Was appropriate statistical analysis used?** | + | + | + | + | + | + | - | + |
| **Quality of the study** | MODERATE QUALITY | MODERATE QUALITY | MODERATE QUALITY | MODERATE QUALITY | HIGH QUALITY | MODERATE QUALITY | MODERATE QUALITY | HIGH QUALITY |

+=Yes; -=No; ?=“Unclear”; N/A: not applicable
